# Non-Microtubular Localizations of Microtubule-Associated Protein 6 (MAP6)

**DOI:** 10.1371/journal.pone.0114905

**Published:** 2014-12-19

**Authors:** Sylvie Gory-Fauré, Vanessa Windscheid, Jacques Brocard, Sylvie Montessuit, Ryouhei Tsutsumi, Eric Denarier, Yuko Fukata, Christophe Bosc, Julie Delaroche, Nora Collomb, Masaki Fukata, Jean-Claude Martinou, Karin Pernet-Gallay, Annie Andrieux

**Affiliations:** 1 Inserm, U836, Physiopathologie du cytosquelette, BP170, Grenoble, France; 2 University Grenoble Alpes, Grenoble Institut des Neurosciences, BP170, Grenoble, France; 3 Department of Cell Biology, University of Geneva, Sciences III, Geneva, Switzerland; 4 Division of Membrane Physiology, Department of Cell Physiology, National Institute for Physiological Sciences, Aichi, Japan; 5 Commissariat à l'énergie atomique, Institut de Recherches en Technologies et Sciences pour le Vivant, Groupe Physiopathologie du Cytosquelette, Grenoble, France; Institute of Molecular and Cell Biology, Biopolis, United States of America

## Abstract

MAP6 proteins (MAP6s), which include MAP6-N (also called Stable Tubule Only Polypeptide, or STOP) and MAP6d1 (MAP6 domain-containing protein 1, also called STOP-Like protein 21 kD, or SL21), bind to and stabilize microtubules. MAP6 deletion in mice severely alters integrated brain functions and is associated with synaptic defects, suggesting that MAP6s may also have alternative cellular roles. MAP6s reportedly associate with the Golgi apparatus through palmitoylation of their N-terminal domain, and specific isoforms have been shown to bind actin. Here, we use heterologous systems to investigate several biochemical properties of MAP6 proteins. We demonstrate that the three N-terminal cysteines of MAP6d1 are palmitoylated by a subset of DHHC-type palmitoylating enzymes. Analysis of the subcellular localization of palmitoylated MAP6d1, including electron microscopic analysis, reveals possible localization to the Golgi and the plasma membrane but no association with the endoplasmic reticulum. Moreover, we observed localization of MAP6d1 to mitochondria, which requires the N-terminus of the protein but does not require palmitoylation. We show that endogenous MAP6d1 localized at mitochondria in mature mice neurons as well as at the outer membrane and in the intermembrane space of purified mouse mitochondria. Last, we found that MAP6d1 can multimerize via a microtubule-binding module. Interestingly, most of these properties of MAP6d1 are shared by MAP6-N. Together, these results describe several properties of MAP6 proteins, including their intercellular localization and multimerization activity, which may be relevant to neuronal differentiation and synaptic functions.

## Introduction

The eukaryotic cytoskeleton, especially the microtubular network, is responsible for cellular morphology, membrane dynamics, intracellular transport, cell division and locomotion. Microtubules are highly dynamic structures composed of αβ-tubulin dimers that switch between growing and shrinking phases [1], [2]. When microtubules are formed with pure tubulin *in vitro*, they disassemble at low temperatures or in the presence of depolymerizing drugs such as nocodazole. Intrinsic microtubule dynamics and stability are tightly regulated in cells by a large number of microtubule-associated proteins (MAPs). In the nervous system, microtubules are key components in the establishment of neuronal polarity, function and signaling. Moreover, cellular microtubules and especially neuronal microtubules resist depolymerizing experimental conditions [3], [4]. This stability has been shown to depend primarily on microtubule association with proteins of the MAP6 family (MAP6s), which include MAP6 (also Stable Tubule Only Polypeptide, or STOP) and MAP6d1 (MAP6 domain-containing protein 1, also called STOP-Like protein 21 KD, or SL21) proteins [5]–[8]. MAP6 proteins are expressed in vertebrates in multiple tissues, including the brain, heart, muscle, kidney, lung and testis [9]. In brain, MAP6 is expressed in many structures, including the olfactory system, cortical layer VII, hippocampus, hypothalamus and cerebellum [10]. At the cellular level, MAP6s have been found in neurons, astrocytes, oligodendrocytes, fibroblasts and pulmonary endothelium [6], [7], [11], [12]. MAP6 proteins are encoded by *Map6* and *Map6d1* genes [5], [13], and MAP6 isoforms are the products of alternatively spliced mRNAs or alternative promoters [9]. The main MAP6 isoforms in the mouse central nervous system are MAP6-E (E-STOP), which is expressed during neurodevelopment and in adult brain, and MAP6-N (N-STOP) and MAP6d1 (SL21), which are expressed postnatally.

MAP6 proteins have been shown to stabilize microtubules (as observed by induction of nocodazole resistance) at physiological temperatures. Microtubule stabilization by MAP6-N is mediated by short repeated sequences called Mn modules [14]. The binding of MAP6-N to microtubules through Mn modules is regulated by Ca^++^/calmodulin and/or phosphorylation [15]. Interestingly, CaMKII phosphorylation of MAP6-N reportedly induces its relocalization toward actin filaments in neurons [15]. MAP6-N binding to microtubules and stabilization of microtubules against cold exposure involve both the Mn modules and other modules called Mc modules [14], [16]. MAP6d1 contains a single Mn module similar to the sequence of the MAP6 Mn3, and it is crucial for microtubule stabilization [5].

MAP6 proteins reportedly associate with the Golgi apparatus through palmitoylation of their N-terminal domains [5]. Palmitoylation is a reversible modification catalyzed by membrane-bound aspartate-histidine-histidine-cysteine (DHHC) palmitoyl acyltransferases. These enzymes represent a large family of at least 23 members exhibiting subcellular and tissue-specific localizations [17], [18]. Palmitoylation usually results in tethering proteins to the cytosolic surfaces of membranes, including the Golgi, endoplasmic reticulum and plasma membranes [17]. Palmitoylation can also regulate protein–protein interactions by controlling the conformation of the modified protein or by spatially coupling protein complexes within lipid microdomains [19].

In this study, we focus on neuronal isoforms of MAP6 proteins (MAP6-N, MAP6-E and MAP6d1). Using ectopic expression of MAP6 proteins (wild type, fragments or mutated forms) in 3T3 cells or in primary cultured neurons, we investigate the several biochemical properties of MAP6 proteins. We demonstrate that the three N-terminal cysteines of MAP6d1 (Cys 5, 10, 11) can be palmitoylated. When expressed in 3T3 cells or in primary neurons, we observed a palmitoylation-dependent association of MAP6d1 with the Golgi apparatus and the plasma membrane. Additionally, we can also observed MAP6d1 interaction with mitochondria via its N-terminal domain, independently of its palmitoylation. Lastly, we show that MAP6d1 can multimerize via its microtubule-binding module Mn. We also provide evidence that the MAP6-N isoform can interact with the Golgi in a palmitoylation-dependent manner and with mitochondria through its N-terminal domain. Together, these results describe several intrinsic properties of MAP6 proteins when transfected in heterologous cells, including several subcellular membranous localization and their ability to multimerize. These intrinsic abilities of MAP6s proteins are clearly under cellular regulations which target MAP6 proteins to one or another subcellular compartment.

## Materials and Methods

### Plasmid constructs

The replacement of Cys5, Cys10 and Cys11 by glycine residues was performed by PCR on the plasmid pSG5-SL21 [5] with Advantage-GC2 Polymerase Mix (BD Biosciences), with a degenerate 5′-oligonucleotide containing a Bgl II extension: 5′-AgA TCT ATg gCg Tgg CCC **ggC** ATC AgC Cgg CTA TgC CTg gCC –3′ for MAP6d1-GCC-myc, 5′-AgA TCT ATg gCg Tgg CCC TgC ATC AgC Cgg CTA **ggC** TgC CTg gCC –3′ for MAP6d1-CGC-myc, 5′-AgA TCT ATg gCg Tgg CCC TgC ATC AgC Cgg CTA TgC **ggC** CTg gCC –3′ for MAP6d1-CCG-myc, 5′-AgA TCT ATg gCg Tgg CCC **ggC** ATC AgC Cgg CTA **ggC** TgC CTg gCC –3′ for MAP6d1-GGC-myc, 5′-AgA TCT ATg gCg Tgg CCC **ggC** ATC AgC Cgg CTA TgC **ggC** CTg gCC –3′ for MAP6d1-GCG-myc, 5′-AgA TCT ATg gCg Tgg CCC TgC ATC AgC Cgg CTA **ggC** CTg gCC –3′ for MAP6d1-CGG-myc. The 5′-oligonucleotides for MAP6d1-CCC-myc, MAP6d1-GGG-myc MAP6d1-ΔMn3-myc and MAP6d1-Δ2-34 were described in [5]. For MAP6[1–41]CCC and MAP6[1–41]GGG constructs, PCR was performed on plasmid pSG5-MAP6-N [8], previously named pSG5-STOP, with 5′-oligonucleotides containing a Nhe I extension 5′-gCT AgC AgA gCC ACC ATg gCg Tgg CCg TgC ATC ACT Agg gCC TgC ATC gCC –3′ and 5′- gCT AgC AgA gCC ACC ATg gCg Tgg CCg **ggC** ATC ACT Agg gCC **ggC** ATC gCC –3′, respectively, and the reverse primer containing a Sfo I extension 5′- ggC gCC Tgg ATg TTC ggT GGC CTC –3′. The resulting PCR products were first cloned into pCR2.1-TOPO (Invitrogen) and subcloned in pcDNA3.1(-)/Myc-His-A vector (Invitrogen) to be fused to DNA encoding the myc epitope. For MAP6d1[1-36]CCC and MAP6d1[1–36]GGG constructs, plasmids MAP6d1-CCC-myc and MAP6d1-GGG-myc were digested with Nde I and Sma I and cloned into pcDNA3.1(−)/Myc-His-A vector digested by Nde I and EcoR V. MAP6-N cDNA [8] and the cDNAs encoding DHHC proteins were cloned in pEF-Bos-HA as previously described [20]. Plasmid MAP6-NΔ2-19, previously named N-STOPΔ2-19, was described in [5]. Plasmids targeting the plasma membrane (pEYFP-Mem) and mitochondria (pDsRed2-Mito) were purchased from Clontech. For yeast two-hybrid experiments, the mouse MAP6d1 coding sequence was cloned into the BamH I site of pLex10 [21] and the Nco I-BamH I sites of pAct2 vector; the control plasmid was pLex10-lamin [22].

### Antibodies

The following primary antibodies were used: rat monoclonal anti-tubulin (YL1/2) diluted to 1/5,000 [23], mouse monoclonal anti-myc tag diluted 1/5000 (Abgent, AM1007a), -GM130 diluted to 1/1,000 (BD Transduction Laboratories, 610823); -OPA1 diluted to 1/1,000 (BD Transduction Laboratories, 612606), goat anti-VDAC diluted to 1/1,000 (Santa Cruz, sc-8828), and rabbit polyclonal anti-Tom20 diluted to 1/1,000 (Santa Cruz, sc-11415), -giantin (Covance, PRB-114C-200) diluted to 1/2,000, -Catalase diluted to 1/1,000 (Abcam, 1871), -SLP2 Ab diluted to 1/500 (home made)[24], -Cytochrome c diluted to 1/2,000 (home made) [25], -MAP6 (23N) diluted to 1/400 (home made) [7], -MAP6d1 (3315) diluted to 1/400 (home made) [5]. Mouse monoclonal antibody, SLF10, was obtained using recombinant GST-MAP6d1 protein as antigen [5] and diluted to 1/4,000 for western blotting. For immunoprecipitation, the following primary antibodies were used: mouse monoclonal anti-MAP6 (mAb 175) diluted to 1/100 [26], -SLF10 diluted to 1/100, - myc-tag diluted to 1/200 [27], rabbit polyclonal antibody against GFP diluted to 1/100 (Life technologies, A-11122). Secondary antibodies were donkey anti-mouse Alexa 488-coupled (Molecular Probes), donkey anti-rabbit Cy3- or Cy5-coupled (Jackson Immuno Research), donkey anti-rat Alexa 488-coupled (Molecular Probes), and HRP-coupled anti-mouse and anti-rabbit (Jackson Immuno Research).

### Cell culture

HEK-293 (ATCC, CRL1573), COS 7 (ATCC, CRL1651) and NIH/3T3 (ATCC, CRL1658) cells were cultured in DMEM-Glutamax supplement with 10% SVF and 1% penicillin/streptomycin (Invitrogen). Hippocampal neuronal cell cultures were prepared as previously described [5].

### Transfection

HEK-293, COS 7 and NIH/3T3 cells were transfected with the cDNAs described above, using Lipofectamine 2000 (Invitrogen) (HEK-293) or the Nucleofector system (Lonza) (COS 7 and NIH/3T3), according to the manufacturer's instructions. Transfected cells were plated either on coverslips for immunofluorescence or in plastic culture dishes for immunoprecipitation and metabolic labeling.

### Immunofluorescence

Six to 18 h after transfection, NIH/3T3 cells were fixed for 25 min with PFS (4% paraformaldehyde, 4% sucrose) and then permeabilized for 3 min with PBS containing 0.2% Triton X-100. Neurons were fixed with PFS 24 h after transfection and permeabilized for 1 min with PBS+0.1% Triton. For endogenous MAP6d1 detection, neurons were fixed for 25 min with PHEM buffer (60 mM PIPES, 25 mM Hepes, 5 mM EGTA, 2 mM MgCl_2_) containing 3.7% paraformaldehyde, 0.025% glutaraldehyde and 3.7% sucrose and permeabilized for 1 min with PBS containing 0.1%. Triton Fixed cells were then incubated with primary antibodies for 45 min in PBS - Tween 0.2% and with secondary antibodies for 40 min. Cells were analyzed with a confocal microscope (LSM 710, Zeiss) or an inverted microscope (Axioscope 50, Zeiss) controlled by Metaview software (Universal Imaging, Downingtown, PA).

### Metabolic labeling

[^3^H] Palmitate labeling and immunoprecipitation were performed as previously described [5], with slight modifications. NIH/3T3 cells were co-transfected with the Nucleofector kit according to the manufacturer's instructions (Lonza), with DHHC-3 cDNA and MAP6d1-myc cDNAs with or without different cysteine mutations. Five hours after transfection, cells were labeled for 3 h with 500 µCi [^3^H]-palmitic acid (Amersham). *In vivo* palmitoylation tests were performed on hippocampal neurons after 28 d of differentiation *in vitro*. Screening of the candidate PATs was performed in HEK-293 cells as previously described [18], [20].

### Immunoprecipitation and western blot analysis

Fifteen hours after transfection, COS cells were washed and scraped with PBS. After centrifugation at 12,000 *g* for 2 min, cells were lysed in IP buffer (50 mM Tris, 150 mM NaCl, 0.05% deoxycholate, 1% Triton X-100, 10% glycerol, pH 8.0) in the presence of protease inhibitors (Complete Cocktail tablets, Roche). After centrifugation of the cell lysate at 12,000 *g* for 5 min at 4°C, the supernatant was incubated for 1 h at 4°C with 5 µl anti-myc, anti-MAP6 mAb175 or anti-GFP antibody pre-incubated for 1 h with 20 µl of Protein G-Sepharose (myc and mAb175) or A-Sepharose (GFP) (Amersham Biosciences). After centrifugation at 4,000 *g* for 2 min, the immunoprecipitates were washed five times in IP buffer for 5 min at 4°C. Samples were separated by SDS-PAGE.

### Electron microscopy

NIH/3T3 cells or neurons were transfected using AMAXA system with MAP6d1-myc and then fixed after 6 h (NIH/3T3) or 48 h (neurons) with 0.1 M phosphate buffer pH 7.2 containing 2% paraformaldehyde and 0.2% glutaraldehyde for 2 h and then washed twice in 0.1 M phosphate buffer and once in 0.1 M phosphate buffer at pH 7.2 with 50 mM glycine. Using a cell scraper, cells were gently detached from the Petri dish, centrifuged and embedded in 10% gelatin. Small pieces of the cell pellet were then cut and incubated for 4 h in 2.3 M sucrose before being frozen in liquid nitrogen. Ultra-thin cryosections of these samples (50 nm) were made at −120°C using an ultra-cryo-microtome (Leica-Reichert, Wetzlar, Germany) and retrieved with a 1∶1 solution of 2.3 M sucrose and 2% methylcellulose according to Liou [28]. For labeling, cryosections were first incubated with primary anti-myc antibody, then incubated with a rabbit anti-mouse bridge (Jackson ImmunoResearch, USA) and revealed with a gold-conjugated protein A (CMC, Utrecht, The Netherlands). Finally, cryosections were stained with 1% uranyl acetate in 2% methylcellulose, and cells were viewed with a transmission electron microscope (1200EX JEOL, Japan) at 80 kV. Images were acquired with a digital camera (Veleta, Olympus, Tokyo, Japan). Immunolocalization of MAP6d1-GFP in neurons was achieved similarly, except that neurons were transfected for 48 h before fixation, and MAP6 d1-GFP was detected with an anti-GFP antibody (Abcam, Cambridge, UK) that was directly revealed with the gold-conjugated protein A.

### Isolation and Purification of Mitochondria

Mouse brains were dissected, rinsed twice with ice-cold MB buffer (Mannitol 210 mM, Sucrose 70 mM, Hepes 10 mM, EDTA 1 mM, pH 7.5) and hand-homogenized in 6 ml of MB using a glass homogenizer. Cells from the homogenate were broken by twenty passages through a 25-G needle fitted on a 10-ml syringe. The resultant homogenate (brain homogenate) was centrifuged at 1,500 *g* for 5 min at 4°C to remove nuclei and unbroken cells. The 1,500 *g* supernatant was centrifuged twice at 6,000 *g* for 10 min to provide a mitochondria-enriched pellet. The supernatant was centrifuged at 100,000 *g* for 30 min to separate the cytosol (supernatant) from various membranes including ER membranes and the Golgi in the pellet (Pellet 100,000 *g*). The mitochondria-enriched pellet was gently resuspended in 2 ml of MB for further purification on a discontinuous sucrose gradient. The gradient was prepared by carefully layering stepwise 15 ml of 1.6 M and 20 ml 1.2 M sucrose solutions in two 35 ml centrifuge tubes. Crude mitochondrial fraction (1 ml) was layered on top of the sucrose cushions and centrifuged at 100,000 *g* for 2 h at 4°C. The mitochondria were collected at the interface of the sucrose cushions after aspiration of the top sucrose layer. They were then resuspended in 4 ml MB and centrifuged twice at 11,700 *g* for 10 min. The mitochondrial pellet was resuspended in 0.2 ml MB, and the protein concentration was assessed using a BioRad Bradford assay.

### Proteinase K (PK) Accessibility Test

The mitochondria obtained after enrichment on a discontinuous sucrose gradient as mentioned above were used for the PK accessibility test. Mitochondria were resuspended at a final concentration of 1 mg/ml in 100 µl MB, swelling buffer (10 mM HEPES-KOH, pH 7.4) or MB+0.2% (v/v) Triton X-100. PK (Roche Diagnostics GmbH) was added to a final concentration of 0.2 µg/ml, and the mixture was incubated on ice for 30 min. PMSF (2 mM) was then added to inhibit the PK. Samples were analyzed by western blotting.

### Yeast two-hybrid experiment

The interactions were tested following standard procedures. Interactions were detected both by growth on agar selection medium lacking leucine, tryptophan and histidine and by staining for β-galactosidase activity. Vector pLex10 containing lamin cDNA was used as control for the specificity of interaction with the pGAD vector.

## Results

MAP6d1 was used as a prototype to investigate the biochemical properties and subcellular localization of MAP6 proteins.

### Palmitoylation of MAP6d1

**Figure 1:**
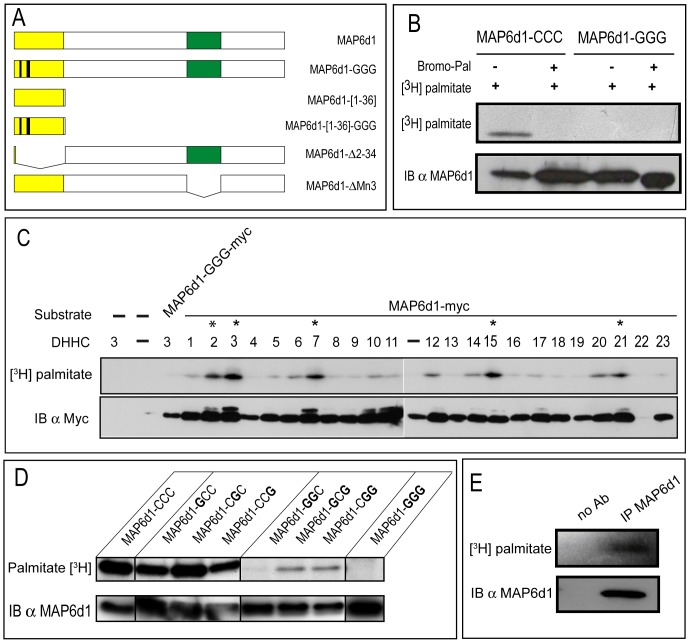
MAP6d1 is palmitoylated on the N-terminal domain. *A*, Schematic representation of MAP6d1 constructs. *B,* NIH/3T3 cells overexpressing MAP6d1 (MAP6d1-CCC) or a MAP6d1 mutant (MAP6d1-GGG) tagged with myc were labeled with [^3^H]-palmitate. The cells were treated with 100 µM 2-bromopalmitate (Bromo-Pal) or vehicle for 3 h. After immunoprecipitation with anti-myc antibody, proteins were separated by SDS-PAGE and subjected either to autoradiography ([^3^H]-palmitate) or to immunoblotting (MAP6d1 mAb SLF10). *C,* Palmitoyl acyltransferase (DHHC) activities on MAP6d1. Each plasmid encoding a DHHC was transfected with the MAP6d1-myc plasmid into HEK-293 cells. After metabolic labeling with [^3^H]-palmitate, proteins were separated by SDS-PAGE followed by autoradiography (*upper panel*) and immunoblotting, using an anti-myc antibody to detect MAP6d1 (*lower panel*). DHHC proteins that enhance MAP6d1 palmitoylation are marked with an asterisk. *D,*MAP6d1 is palmitoylated on Cys 5, 10 and 11 residues. NIH/3T3 cells were transfected with a plasmid encoding MAP6d1-myc (MAP6d1-CCC) or mutant forms in which one, two or three cysteines were replaced with glycine: MAP6d1-GCC-myc, MAP6d1-CGC-Myc, MAP6d1-CCG-Myc, MAP6d1-GGC-Myc, MAP6d1-GCG-Myc, MAP6d1-CGG-Myc, and MAP6d1-GGG-Myc. Cell extracts were processed as in B. Mutating all 3 MAP6d1 cysteines (MAP6d1-GGG) abolished palmitoylation. *E*, MAP6d1 protein palmitoylation *in vivo:* cultured mouse hippocampal neurons (28 days of DIV) were labeled with [^3^H]-palmitate. MAP6d1 proteins were immunoprecipitated from total cell lysates with MAP6d1 antibody (mAb SLF10). Immunoprecipitated proteins were separated by SDS-PAGE and subjected to autoradiography ([^3^H]-palmitate) *IB*: immunoblot, *IP*: immunoprecipitation, *Bromo-Pal*: 2-bromopalmitate.

To analyze the palmitoylation of MAP6d1 proteins, we constructed expression vectors encoding MAP6d1 or mutant forms of MAP6d1 (Fig. 1*A*) in which cysteines 5, 10 and 11 were replaced with glycine (MAP6d1-GGG). These cDNA constructs were transfected into NIH/3T3 cells. After incubation with [^3^H]-palmitate, MAP6d1 proteins were immunoprecipitated from cell extracts and separated by SDS-PAGE for analysis by autoradiography ([^3^H]-palmitate) and immunoblotting (Fig. 1*B*). A radioactive band co-migrated with MAP6d1, demonstrating that MAP6d1 is palmitoylated in cells. Importantly, MAP6d1 palmitoylation was not observed in the presence of the palmitoylation inhibitor 2-bromopalmitate (Bromo-Pal) and did not occur when cysteines 5, 10 and 11 were mutated into glycines.

Palmitoylation occurs *in vivo* through palmitoyl acyltransferases, which belong to a family of 23 enzymes containing a conserved DHHC motif [18]. We thus tested whether any of these 23 DHHCs palmitoylate MAP6d1. HEK-293 cells were co-transfected with MAP6d1 plasmid and each of the 23 DHHC-encoding plasmids. After incubation with [^3^H]-palmitate, the cell extracts were analyzed by autoradiography ([^3^H]-palmitate) and immunoblotting. As shown in Fig. 1*C*, MAP6d1 palmitoylation was markedly enhanced when DHHCs 2, 3, 7, 15 and 21 were co-expressed. Interestingly, the genes encoding these DHHCs are located on common branches and sub-branches in the DHHC phylogenetic tree [29], [30]. In addition, DHHCs 6, 10, 12, 14 and 20 also showed palmitoyl transferase activity.

To determine which MAP6d1 cysteine residues are palmitoylated, we designed a series of mutants in which one, two or all three of the cysteines were converted into glycine. MAP6d1 or mutants forms were expressed in NIH/3T3 cells, and palmitoylation was assessed in the presence of DHHC3 (Fig. 1*D*). Mutants containing only cysteine 5, 10 or 11 incorporated palmitate, but the mutant containing only Cys11 incorporated a very limited amount of palmitate (Fig. 1*D*). All the mutants containing two cysteine residues incorporated large amounts of palmitate (Fig. 1*D*), suggesting that the presence of two cysteine residues enables cooperative palmitate transfer. The control mutant MAP6d1-GGG, with no N-terminal cysteines, did not incorporate palmitate (Fig. 1*D*).

Finally, we investigated the palmitoylated forms of MAP6d1 *in vivo.* Cultured mature mouse hippocampal neurons were incubated with [^3^H]-palmitate, and MAP6d1 protein was immunoprecipitated from total cell lysates and analyzed by western blotting and autoradiography (Fig. 1*E*). A radioactive band co-migrated with MAP6d1, demonstrating MAP6d1 palmitoylation under physiological conditions.

### MAP6d1 can interact with the plasma membrane and mitochondria

**Figure 2:**
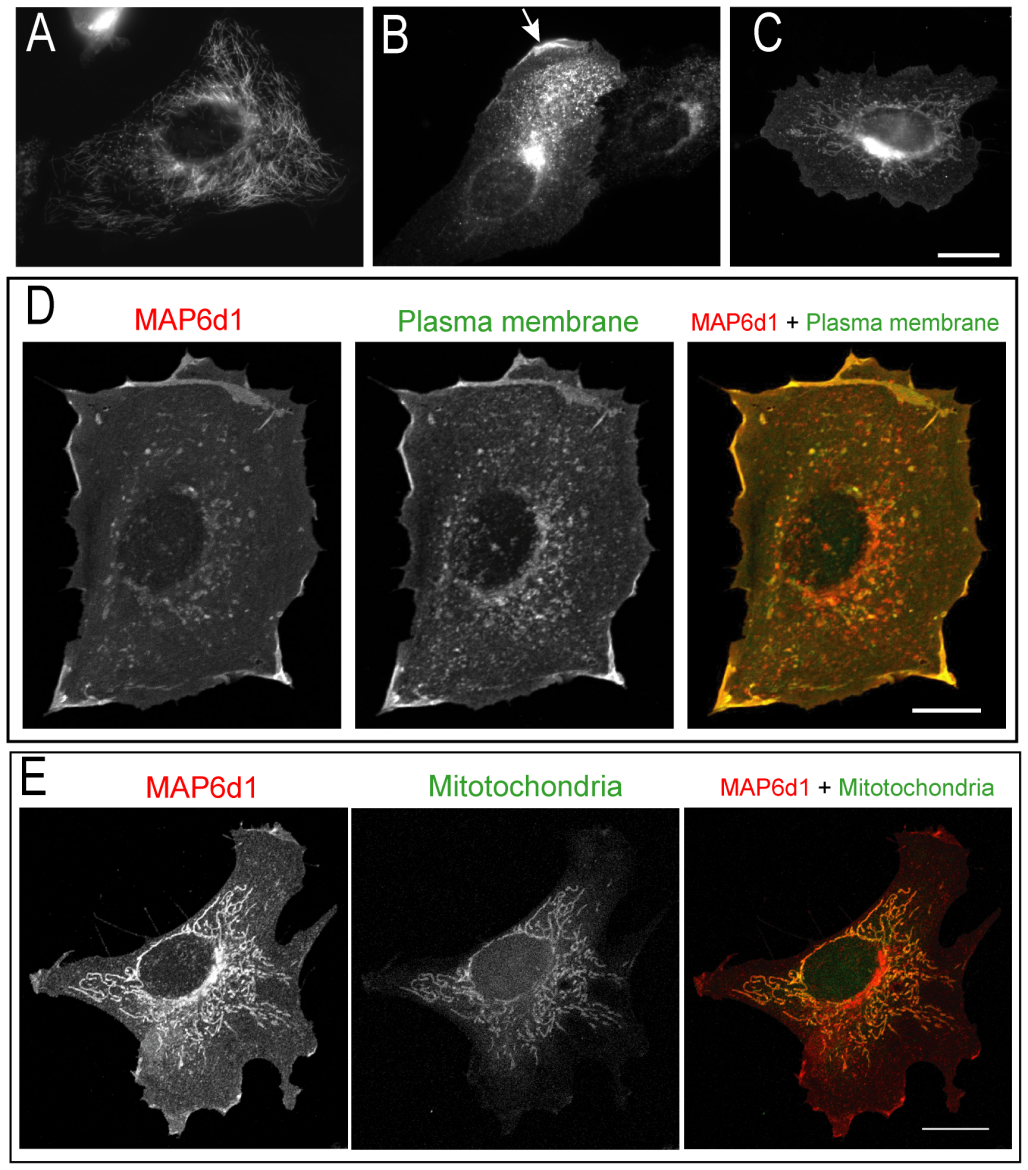
MAP6d1 can localize to the plasma membrane and mitochondria. *A*–*C,* NIH/3T3 cells transfected with a MAP6d1-myc encoding plasmid were fixed with PFA-sucrose and immunolabeled for MAP6d1 (mAb anti-myc). Localization at microtubules (A), Golgi (B), plasma membrane (B, arrow), mitochondria (C) can be observed. *D*–*E,* NIH/3T3 cells were transfected with a plasmid encoding MAP6d1-myc and fluorescent markers for the plasma membrane (*D*) or mitochondria (*E*). After fixation and immunolabeling MAP6d1 (mAb anti-myc), cells were analyzed by confocal. MAP6d1 co-localizes with the plasma membrane marker pEYFP-Mem (D) and the mitochondria marker pDsRed2-Mito (*E*). *Bars,* 10 µm.

**Figure 3:**
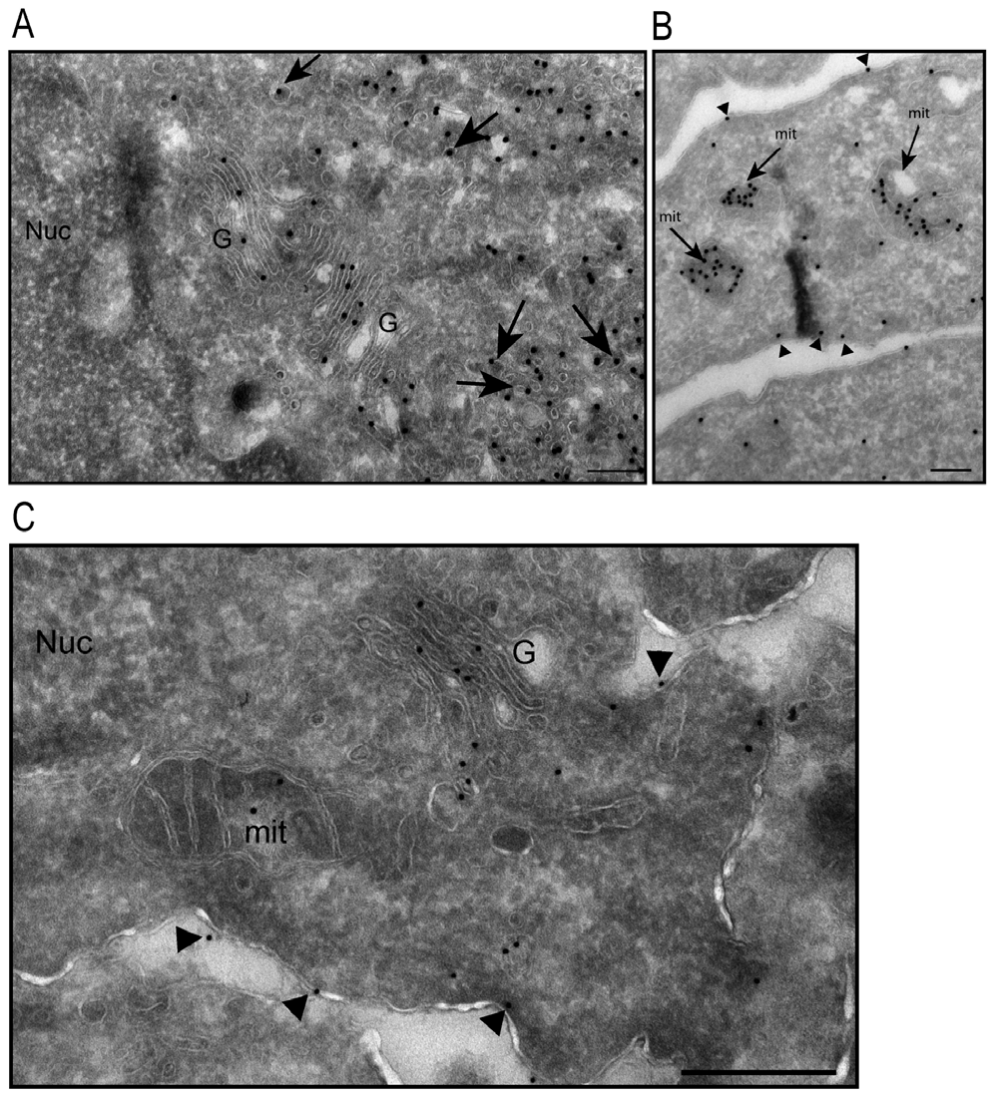
Analysis of MAP6d1 localization by electron microscopy. *A*–*B*, NIH/3T3 cells were transfected with a MAP6d1-myc encoding plasmid and analyzed by electron microscopy. Cryosections were labeled with immuno-gold (mAb anti-myc). MAP6d1 was observed at the Golgi apparatus and in vesicles that are frequently clustered in the cytoplasm (*A, arrows*). MAP6d1 also localized to the plasma membrane (*B, arrowheads*) and within mitochondria (*B, arrows*). *C,* hippocampal neurons were transfected with a plasmid encoding MAP6d1-GFP and immuno-gold localization was performed using an anti-GFP antibody. Localization at the Golgi, mitochondria and plasma membrane (arrowheads) can be observed. No gold particles were found in the nucleus. *Bars,* 200 nm. *G:* Golgi apparatus; *Nuc:* nucleus; *ER:* endoplasmic reticulum.

Palmitoylation of proteins is known to target several membranous compartments including the Golgi, plasma membrane and endoplasmic reticulum [17]. We thus carefully investigated the potential subcellular localization of MAP6d1 in transfected NIH/3T3 cells. As previously published, using methanol fixation, we observed that MAP6d1 was localized at the Golgi and/or on microtubules [5]. Because PFA-sucrose fixation is known to protect membrane compartments better than methanol, we used this procedure to investigate other possible membranous associations of MAP6d1. Interestingly, in some cells transfected with MAP6d1, in addition to microtubules (Fig. 2*A* *)* and Golgi (Fig. 2*B*–*C*), we observed localization at plasma membrane (Fig 2*B*, arrow) and mitochondria (Fig. 2*C*). To confirm MAP6d1 localizations, we transfected cells with MAP6d1 and a plasma membrane marker (pEYFP-Mem) or a mitochondrial marker (pDsRed2-Mito). Using confocal microscopy, we observed co-localization of MAP6d1 with the plasma membrane and mitochondria markers (Fig. 2*D*–*E*). We then performed a quantitative analysis of MAP6d1 distribution at microtubules, Golgi, plasma membrane and mitochondria 24 h after transfection. MAP6d1 localized at microtubules, Golgi, plasma membrane and mitochondria in 3%, 85%, 5.5% and 61% of transfected cells, respectively (Table 1). In some cells, several distributions can be observed (e.g., microtubules and Golgi, Golgi and mitochondria). These results revealed two new localizations of MAP6d1: plasma membrane and mitochondria. We then directly visualized the association of MAP6d1 with internal membranes by analyzing transfected NIH/3T3 cells by electron microscopy. As expected, MAP6d1 immuno-gold labeling (black dots) was associated with the Golgi apparatus in most cells (Fig. 3*A*). MAP6d1 was also associated with the plasma membrane (Fig. 3*B*, *arrowheads*) or with mitochondria (Fig. 3*B*, *arrows*). Additionally, we observed an accumulation of vesicles positively stained for MAP6d1 in many cells (Fig. 3*A*, *arrows*). Note that no positive signal for MAP6d1 was found within the nucleus (Fig. 3*A*) or the endoplasmic reticulum. The protocol designed for electron microscopy did not preserve microtubules, preventing the observation of MAP6d1 on microtubules. These results clearly identified MAP6d1 at the Golgi, plasma membrane and mitochondria in transfected NIH/3T3 cells. We then wondered whether similar localizations could be observed in a neuronal context. For this purpose, primary mouse cultured neurons were transfected with MAP6d1 plasmid. In neurons, electron microscopy revealed MAP6d1 association with the Golgi, plasma membrane (arrows) and mitochondria (Fig. 3*C*), as in NIH/3T3 cells. Altogether, these results clearly indicate the ability of MAP6d1 to interact with several subcellular membranous compartments including Golgi, plasma membrane and mitochondria, each localization being under specific cellular signaling.

**Table 1:** Subcellular localization of MAP6d1 and MAP6d1 mutants.

Constructs	% cells with microtubule localization	% cells with Golgi localization	% cells with plasma membrane localization	% cells with mitochondrialocalization	% cells with other localization
MAP6d1	3	85	5.5	61	4.5
MAP6d1-GGG	13	0	0	60	27
MAP6d1-Δ2-34	18.5	0	0	0	81.5

NIH/3T3 cells were transfected with MAP6d1-myc or MAP6d1-myc mutants for 6 h, fixed using PFA-sucrose and immunolabeled with anti-myc antibody. At least 200 cells were analyzed and for each cell, the localization of MAP6d1 to microtubules, Golgi, plasma membrane and mitochondria has been determined. If no clear localization to one of these four subcellular compartments was found, the cell was quantified as “other”. Please note that there is more than one localization for MAP6d1 in some cells (as illustrated in Fig. 2*A*–*C*), such as Golgi and mitochondria or Golgi and microtubules.

### MAP6d1 palmitoylation is required for interaction with the plasma membrane, not the mitochondria

**Figure 4:**
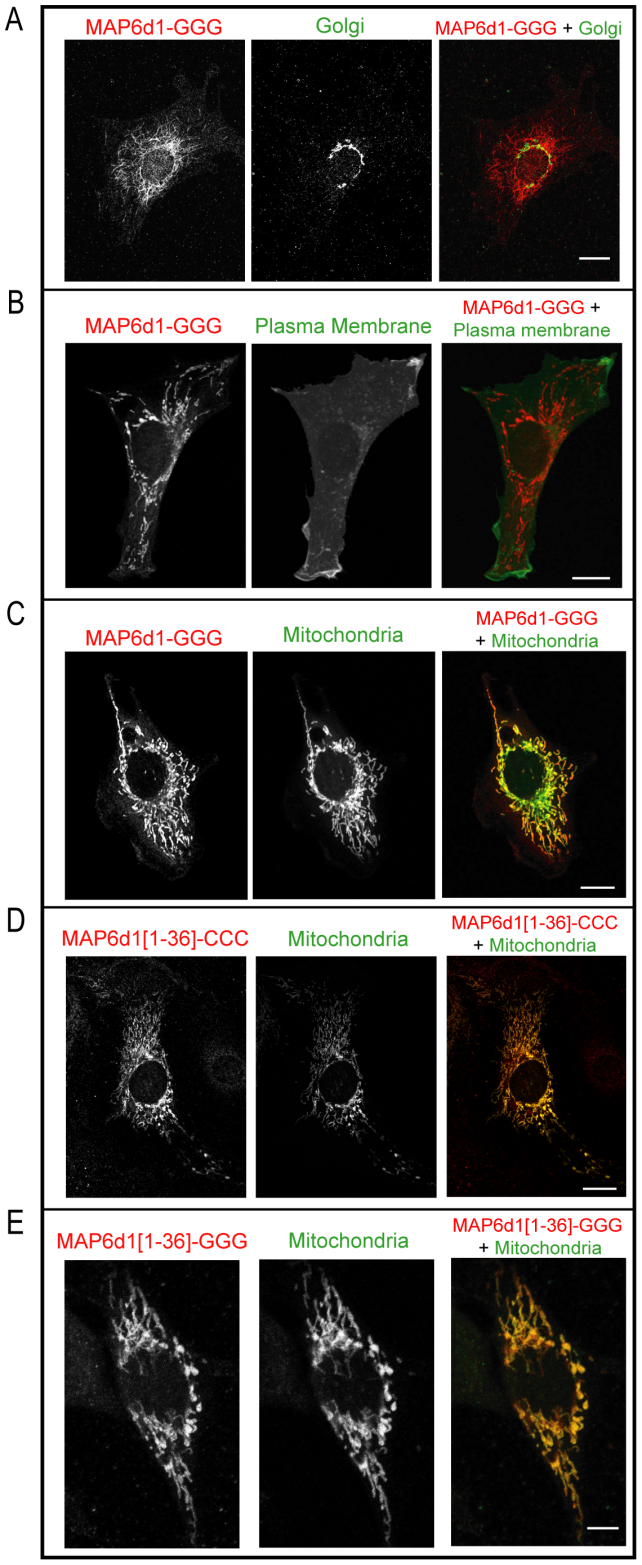
MAP6d1-GGG localizes to the mitochondria but not to the plasma membrane. *A*, NIH/3T3 cells transfected with a plasmid encoding MAP6d1-GGG-myc and GM130 (Golgi marker) antibodies. No colocalization was observed. *B*–*C,* NIH/3T3 cells were transfected with plasmids encoding MAP6d1-GGG-myc and fluorescent markers for the plasma membrane (*B*) or mitochondria (*C*). After fixation and immunolabeling for MAP6d1-GGG, the cells were analyzed by confocal microscopy. *B,* No co-localization of MAP6d1-GGG and the plasma membrane marker was observed. *C,* Co-localization of MAP6d1-GGG and the mitochondrial marker. *D*–*E,* NIH/3T3 cells transfected with plasmids encoding MAP6d1[1–36]-CCC-myc or MAP6d1[1–36]-GGG-myc and fluorescent markers for mitochondria. Both the MAP6d1[1–36]-CCC (*D*) and MAP6d1[1–36]-GGG *(E)* fragments co-localize with the mitochondrial marker. Note that images (*A*–*E*) correspond to a single focal plane through organelle under study and thus these focal planes do not reflect the whole localization of MAP6d1. *Bars,* 10 µm for *A*–*D* and 5 µm for *E*.

Because palmitoylation was required for the interaction between MAP6d1 and the Golgi [5], we investigated whether palmitoylation was also required for its localization to the plasma membrane or mitochondria. The MAP6d1-GGG mutant, which cannot be palmitoylated, did not associate with the Golgi apparatus (Fig. 4*A*) or with the plasma membrane (Fig. 4*B*) but still localized to the mitochondria (Fig. 4*C*). The localization of MAP6d1-GGG mutant was quantified and we found localization at microtubules and mitochondria in 13% and 60% of cells, respectively (Table 1). These results clearly indicate that mitochondrial localization does not require N-terminal cysteines.

Analysis of MAP6d1 putative subcellular localization using the PSORT II program (http://psort.hgc.jp/form2.html) [31] indicates a 48% probability for the protein to be targeted to the mitochondria. This program examines the first 20 aa of a protein and does not consider modifications such as palmitoylation. To determine whether the N-terminus of MAP6d1 is sufficient for targeting to mitochondria, we built a construct encoding aa 1–36 of MAP6d1 (MAP6d1[1–36]-myc, Fig. 1*A*), a domain shared with MAP6. After transfection, MAP6d1[1–36]-myc associated with mitochondria in NIH/3T3 cells (Fig. 4*D*). As expected, mutating the three cysteine residues (MAP6d1[1–36]-GGG) did not affect mitochondrial localization (Fig. 4*E* *)*. Conversely, a MAP6d1 mutant lacking the 34 N-terminal aa (MAP6d1-Δ2-34, Fig. 1*A*) never associated with the mitochondria in NIH/3T3 cells, nor with the Golgi or plasma membrane (Table 1). These results clearly demonstrate that the N-terminus of MAP6d1 directs its localization to the mitochondria independently of its palmitoylation.

**Figure 5:**
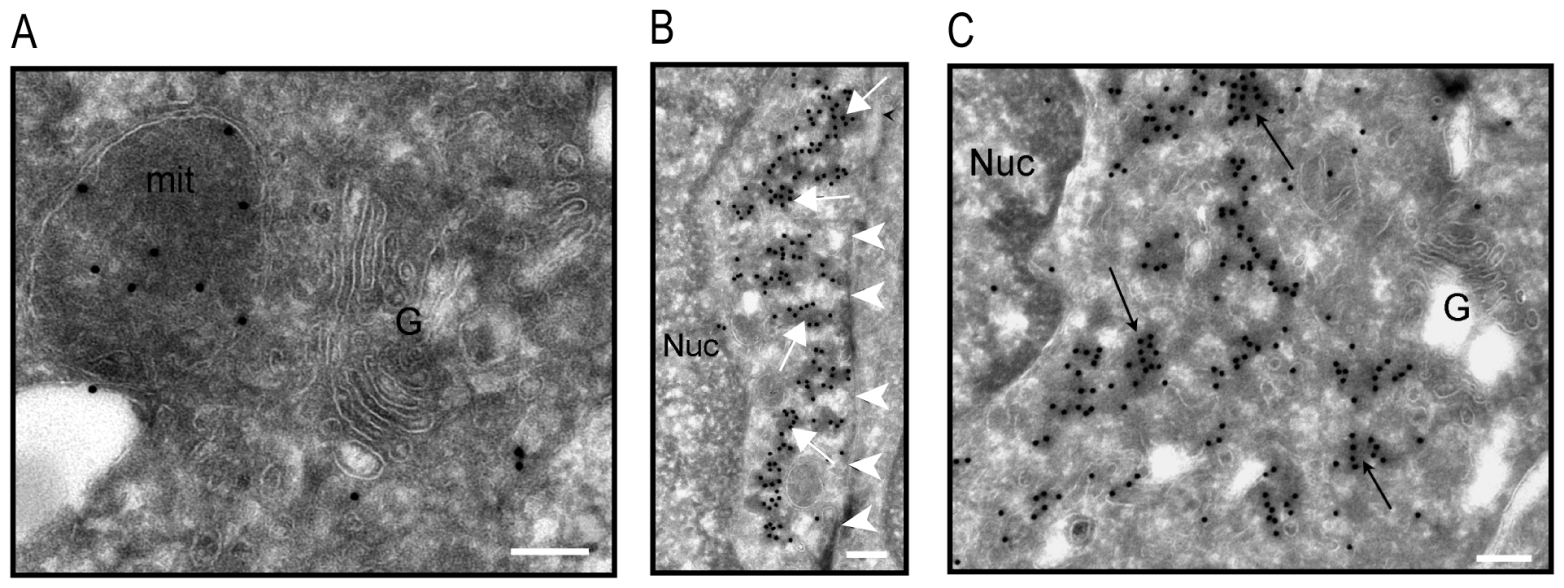
Analysis of MAP6d1-GGG localization by electron microscopy. *A*–*C,* NIH/3T3 cells were transfected with a plasmid encoding MAP6d1-GGG-myc and immuno-gold labeled. Analysis by electron microscopy indicated that MAP6d1-GGG did not associate with the Golgi apparatus (*A,C*) or the plasma membrane *(B, arrowheads)*. In contrast, MAP6d1-GGG was observed within mitochondria (*A*). In addition, strong labeling was observed in electron-dense protein aggregates (arrows, *B*–*C*) that were not associated with vesicles. *G:* Golgi apparatus; *Nuc:* nucleus; *mit:* mitochondria. *Bars*, 200 nm.

To directly visualize the subcellular localization of MAP6d1-GGG, we analyzed MAP6d1-GGG transfected NIH/3T3 cells by electron microscopy. The MAP6d1-GGG mutant did not localize to the Golgi (Fig. 5*A,C*) or the plasma membrane (Fig. 5*B* *, arrowheads*) but was observed in the mitochondria (Fig. 5*A*). The MAP6d1-GGG mutant was also observed as aggregates (Fig. 5*B*–*C* *, arrows*) in the cytoplasm; however, in sharp contrast with the wild-type MAP6d1 (*Fig. 3A*), these aggregates were devoid of membranes. This electron microscopy analysis raises the possibility that MAP6d1 can oligomerize and form aggregates (see below).

### MAP6d1 is associated with mitochondria *in vivo*

**Figure 6:**
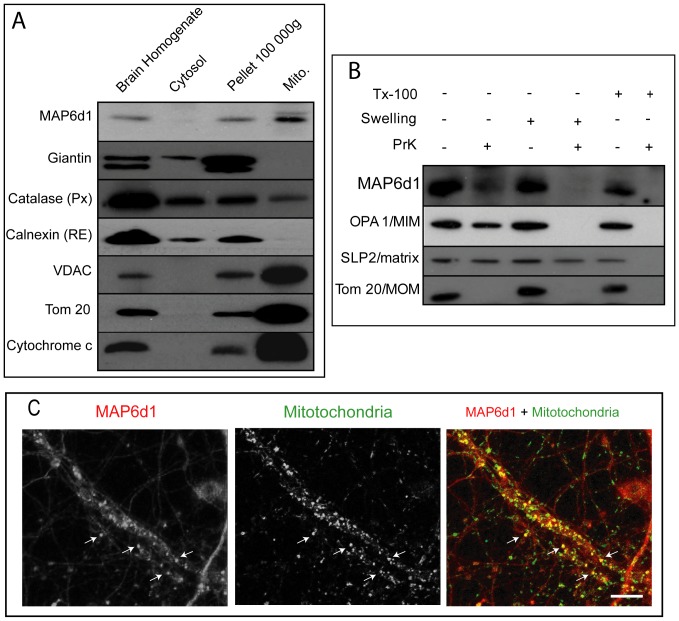
Endogenous MAP6d1 is present in mitochondria. *A,* Proteins from brain homogenate, cytosol, pellet and purified mitochondria (10 µg) were separated by SDS-PAGE and immunoblotted using MAP6d1 and control antibodies against Giantin, catalase, calnexin, VDAC, Tom 20 and Cytochrome C. *B,* Immunoblot analysis of mitochondrial extract after Proteinase K accessibility test. *PrK*, Proteinase K; *Tx-100*, Triton X-100. OPA1 is a mitochondrial intermembrane space protein anchored to the inner membrane (MIM); Tom20 is a mitochondrial outer-membrane protein (MOM); SLP2 is a mitochondrial matrix protein (matrix). MAP6d1 was localized at the outer membrane and in the intermembrane space. *C*, Hippocampal neurons after 14 days of DIV were labeled with anti-MAP6d1 (SLF10) and anti-Tom20 to stain mitochondria. Localization of MAP6d1 at mitochondria can be observed (arrow). Note that these images correspond to a single focal plane. *Bar:* 10 µm.

To assay MAP6d1 association with mitochondria *in vivo*, we performed experiments using purified mitochondria from mouse brain homogenate [32]. The mitochondrial fraction was negative for giantin and calnexin proteins, Golgi and reticulum endoplasmic markers, respectively (Fig. 6*A*) and positive for the mitochondrial markers VDAC, Tom 20 and Cytochrome c (Fig. 6*A*). MAP6d1 was clearly present in the mitochondrial fraction (Fig. *6A*). To further analyze the association of MAP6d1 with mitochondria, we performed a proteinase K accessibility test with or without swelling. Without swelling, the mitochondrial outer-membrane (MOM) proteins such as Tom20 were absent after treatment of mitochondria with proteinase K, whereas the proteins of the mitochondrial intermembrane (MIM) space anchored to the inner membrane such as OPA1 were resistant to the treatment (Fig. 6*B*). When proteinase K treatment was preceded by a swelling step, only mitochondrial matrix proteins such as SLP2 resisted the treatment (Fig. 6*B*). Concerning MAP6d1, when mitochondria were treated with proteinase K without swelling, a fraction of MAP6d1 was protected (Fig. 6*B* *)*, consistent with MAP6d1 localization at the outer membrane and in the intermembrane space. These results were consistent with the electron microscopy images in which MAP6d1 can be observed inside the mitochondria. To directly visualized mitochondria localization of endogenous MAP6d1 in neurons, we performed immunofluorescence on neurons after 14 days of differentiation *in vitro*. Using confocal microscopy, we could observe partial co-localization of MAP6d1 with the mitochondrial markers Tom20 (Fig. 6*C* *, arrows*). All these results show that an endogenous MAP6d1 fraction is targeted to brain mitochondria.

### MAP6d1 can form non-covalent multimers

**Figure 7:**
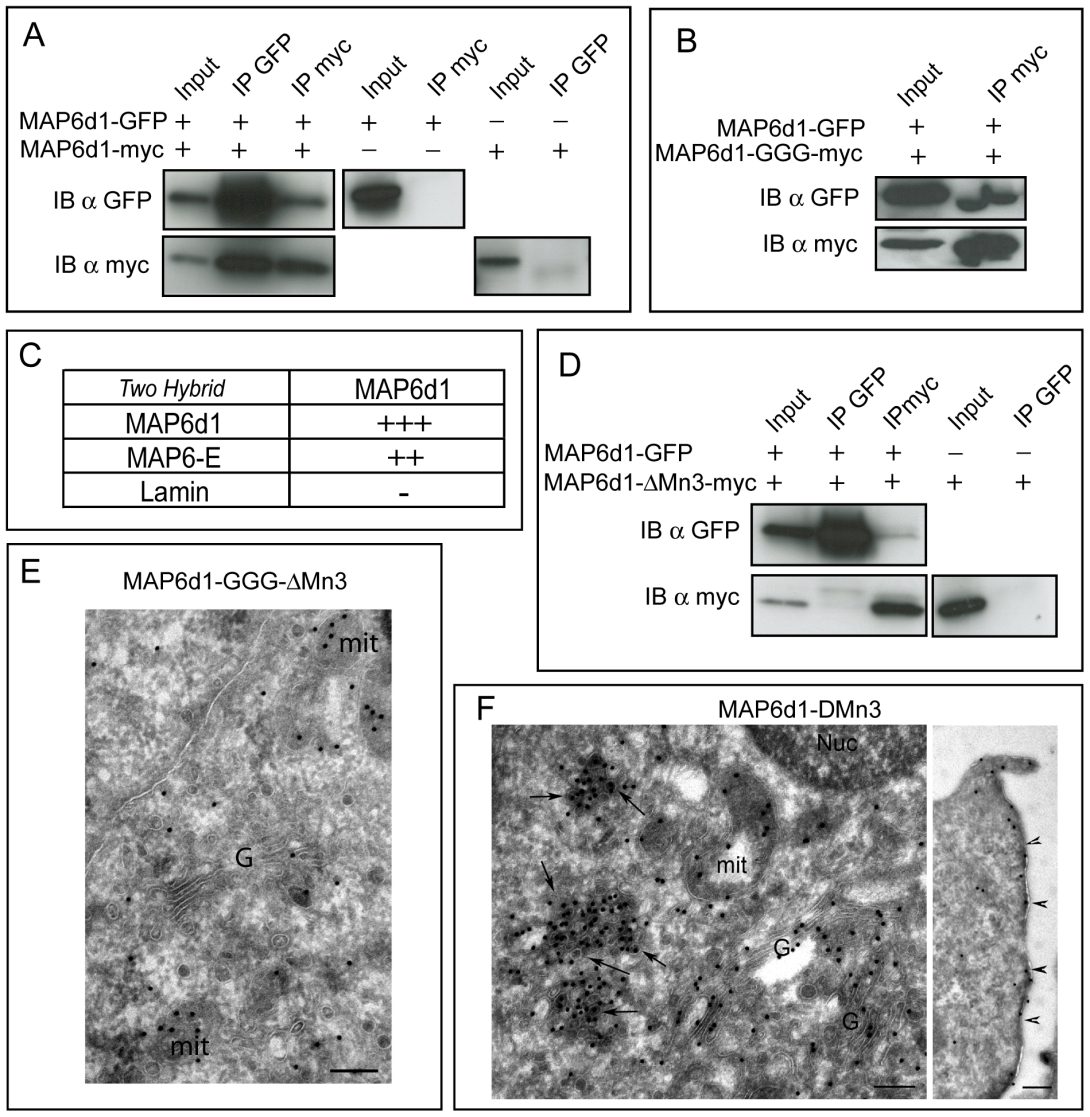
MAP6d1 can multimerize. *A*, COS-7 cells overexpressing MAP6d1-GFP and MAP6d1-myc were lysed. Proteins were immunoprecipitated with antibodies against either GFP or myc, separated by SDS-PAGE and immunoblotted with each antibody. In the control experiments, COS-7 cells over-expressing either MAP6d1-myc or MAP6d1-GFP were lysed, and protein was immunoprecipitated with anti-GFP or anti-myc antibodies, respectively. *B*, MAP6d1 multimerization does not require cysteines. COS-7 cells overexpressing MAP6d1-GFP and MAP6d1-GGG-myc were lysed, and the proteins were immunoprecipitated as in *A.* Both proteins are present in the immunoprecipitate. *C,* Two-hybrid experiments to test the interactions between MAP6d1 and itself, MAP6-E or lamin. MAP6d1 interacts with itself and with MAP6-E. *D,* MAP6d1 multimerization requires the Mn3 module. COS-7 cells overexpressing MAP6d1-GFP and MAP6d1-ΔMn3-myc were lysed, and proteins were immunoprecipitated as in *A.* No co-immunoprecipitation was observed between MAP6d1 and MAP6d1-ΔMn3. *E,* NIH/3T3 cells were transfected with a plasmid encoding MAP6d1-GGG-ΔMn3-myc, and cryosections were labeled using an anti-myc antibody and immuno-gold. MAP6d1-GGG-ΔMn3 is not concentrated on the Golgi apparatus but is dispersed throughout the cytoplasm. Mitochondria are still heavily labeled, but no protein aggregates were observed. *F*, NIH/3T3 cells were transfected with a plasmid encoding the mutant MAP6d1-ΔMn3-myc and analysed as in *E*. MAP6d1-ΔMn3 associates with the Golgi, the plasma membrane (*arrowheads*) and cytoplasmic vesicles (*arrows*). *G:* Golgi apparatus; *mit:* mitochondria. *Bars*, 200 nm.

To investigate whether MAP6d1 multimerizes, we constructed expression vectors encoding MAP6d1 fused to either a myc epitope or GFP at the C-terminus. These proteins were co-expressed in COS-7 cells, immunoprecipitated from cell extracts and analyzed by western blot. We readily detected formation of MAP6d1 multimers in the co-transfected cells, *i.e.*, when MAP6d1 was immunoprecipitated using a myc antibody, we detected MAP6d1-GFP in the immunoprecipitate and *vice versa* (Fig. 7*A*). Similar experiments with MAP6d1-GFP and the MAP6d1-GGG-myc mutant also showed multimerization (Fig. 7*B*), indicating that the N-terminal cysteines are not required for multimerization. To determine whether the observed multimerization is the result of a direct interaction between MAP6d1 molecules, we performed a yeast two-hybrid experiment. This system generally requires direct physical interaction between the bait and target proteins to yield a positive signal. When LexA-MAP6d1 was used as the bait, we detected interaction with GAD-MAP6d1, indicating direct binding. Interestingly, we also found that MAP6d1 can interact with MAP6-E (Fig. 7*C*). We found no interaction between MAP6d1 and the control, lamin (Fig. 7*C*). Together, these results revealed the intrinsic ability of MAPd1 to form homodimers or heterodimers with MAP6-E. Multimerization often involves matching homologous sequences in the proteins. MAP6d1 and MAP6-E share two stretches of homologous sequences: their 35 aa N-terminal domain and their 24 aa Mn3 microtubule-binding module [5]. Because the N-terminal cysteines of MAP6d1 are not involved in multimerization (Fig. 7*B*), we tested whether the Mn3 module is involved. As shown in Fig. 7*D*, a MAP6d1 mutant lacking the Mn3 module (MAP6d1-ΔMn3-myc) was unable to multimerize. These results indicate that MAP6d1 multimerization is independent of palmitoylation but requires the Mn3 microtubule-binding domain. Accordingly, the MAP6d1-GGG-ΔMn3 double mutant (Fig. *7E*) never forms the cytoplasmic aggregates observed in NIH/3T3 cells with the MAP6d1-GGG mutant (Fig. 5*B-C*) but only displays dispersed cytoplasmic labeling as observed by electron microscopy. Note that mitochondrial labeling was still observed (Fig. 7*E*). In the control experiments, we showed that MAP6d1-ΔMn3 associates with the Golgi (Fig. 7*F*), the plasma membrane (Fig. 7*F*, *arrowheads*) and cytoplasmic vesicles (Fig. 7*F* *, arrows*). These results strongly indicate that the Mn3 sequence is involved in multimerization (see the cytoplasmic aggregates devoid of membranes in Fig. 5*B*–*C*) and is dispensable for membrane interactions.

### MAP6-N exhibits properties similar to those of MAP6d1

**Figure 8:**
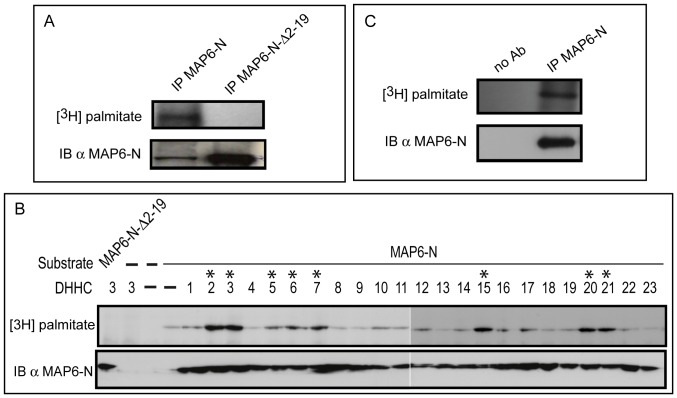
MAP6-N is palmitoylated on the N-terminal domain. *A*, NIH/3T3 cells overexpressing MAP6-N or the MAP6-N mutant lacking the 19 N-terminal aa (MAP6-N-Δ2-19) were labeled with [^3^H]-palmitate. MAP6 proteins were immunoprecipitated from total cell lysate with mAb 175 antibodies. Immunoprecipitated proteins were separated by SDS-PAGE and subjected to autoradiography ([^3^H]-palmitate) and to immunoblotting (23N antibody). *B,* Palmitoyl acyltransferase (DHHC) activities on MAP6-N. Experiments were performed as described in Fig. 1C. *C*, Cultured mouse hippocampal neurons (28 days of DIV) were labeled with [^3^H]-palmitate. MAP6 proteins were immunoprecipitated from total cell lysate with mAb 175 antibodies. Immunoprecipitated proteins were separated by SDS-PAGE and subjected to autoradiography ([^3^H]-palmitate) and to immunoblotting (23N antibody). No-antibody controls were tested by immunoprecipitation.

We examined whether MAP6d1 properties are also exhibited by MAP6-N. To analyze MAP6-N palmitoylation, we constructed expression vectors encoding MAP6 or MAP6 lacking its N-terminal domain (MAP6-N-Δ2-19). As demonstrated for MAP6d1, MAP6-N was palmitoylated in cells. However, the palmitoylation was abolished in the mutant lacking the 19 N-terminal aa (containing the cysteines 5, 10 and 11) (Fig. 8A). We found that a very similar set of DHHCs palmitoylated MAP6-N and MAP6d1, and DHHCs 5, 6 and 20 were additional enzymes that acted on MAP6 (Fig. 8*B*). Finally, we found that endogenous MAP6 is palmitoylated in cultured hippocampal neurons (Fig. 8*C*).

**Figure 9:**
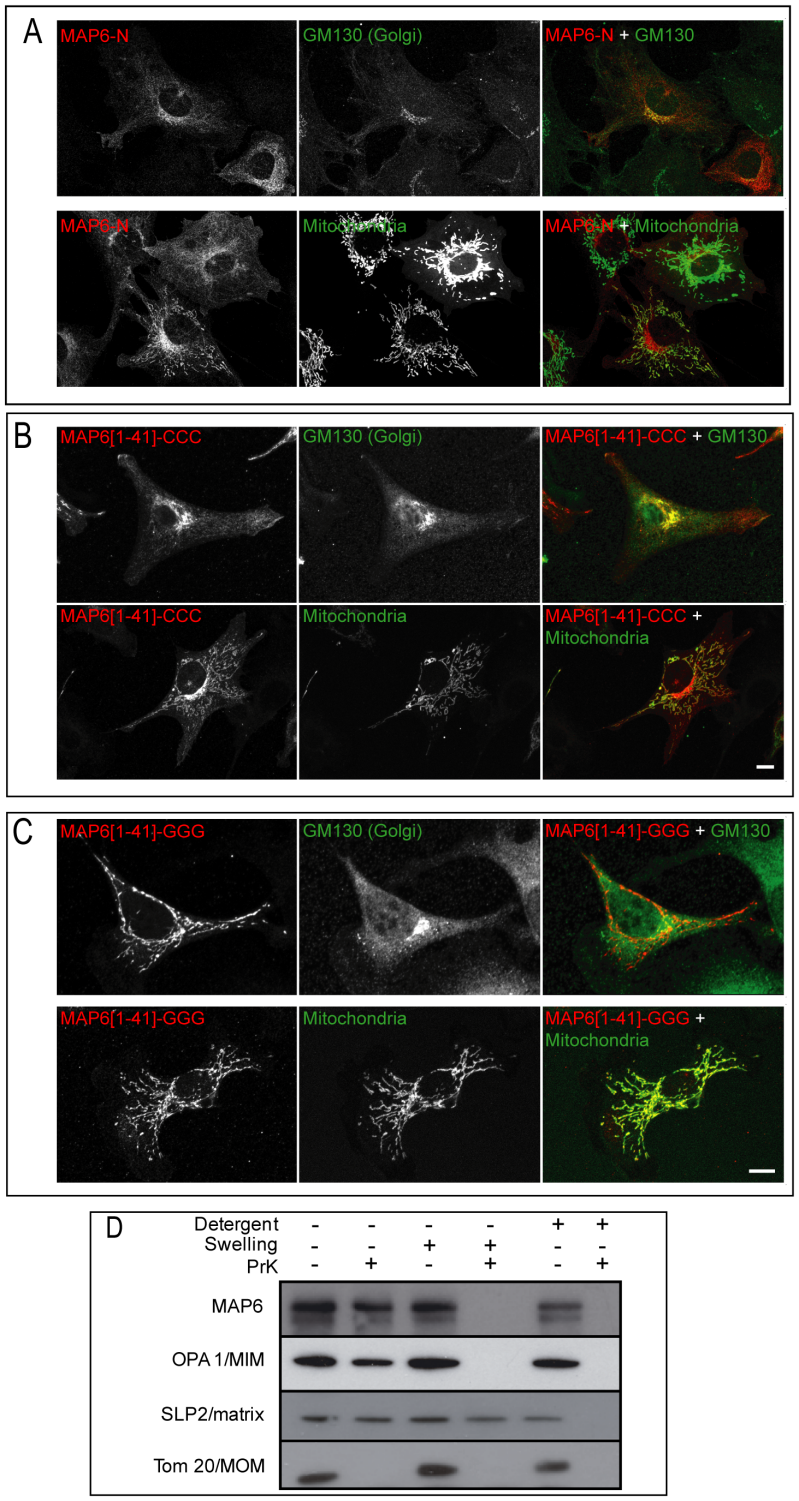
Subcellular localization of MAP6-N. *A*, NIH/3T3 cells were transfected with plasmids encoding MAP6-N-myc and fluorescent markers for Golgi or mitochondria. After fixation and immunolabeling of MAP6-N (mAb anti-myc), the cells were analyzed by confocal microscopy. *A,* Co-localization of MAP6-N with the Golgi marker and partial co-localization with the mitochondria marker. (*B, C*) NIH/3T3 cells overexpressing the myc-tagged MAP6-N fragment corresponding to residues 1 to 41 without (B) or with mutation of the cysteines 5, 10 and 11 *(C)*. After fixation and immunolabeling the MAP6-N fragments (mAb anti-myc), cells were analyzed by confocal microscopy. The MAP6[1]–[41]-CCC fragment localized with both the Golgi and the mitochondria markers, whereas the MAP6[1]–[41]-GGG fragment only localized to the mitochondria. *Bars,* 10 µm. *D,* MAP6d1 is present in purified mitochondria fractions from brain. Mitochondrial localization of MAP6-N was analysed as described in Fig. 6.

MAP6-N transfected into NIH/3T3 cells localized to the Golgi apparatus (Fig. 9*A*). Like MAP6d1, MAP6-N can also be directed to the mitochondria (Fig. 9*A*). Moreover, a minimal fragment corresponding to the N-terminus of MAP6-N (MAP6-[1–41]-CCC) localized to the Golgi and the mitochondria (Fig. 9*B*). Further, mutating the N-terminal cysteines of this minimal fragment to glycines (MAP6-[1–41]-GGG) prevented Golgi localization but not mitochondrial targeting (Fig. 9*C*). Finally, using purified mitochondria from mouse brain tissue, we observed that MAP6-N, as MAP6d1, was associated with mitochondrial fractions *in vivo* (Fig. 9*D*) and localized at the outer membrane and in the intermembrane space as revealed by the proteinase K accessibility test (Fig. 9*D*).

## Discussion

MAP6 proteins stabilize microtubules under depolymerizing experimental conditions such as cold exposure or nocodazole treatment [5]. In addition to binding microtubules, MAP6-N protein have been shown in neurons to interact with the actin cytoskeleton upon phosphorylation by CaMKII [15]. Interaction with both the microtubule and actin cytoskeletons is a shared property of MAP6 proteins and other structural MAPs, including Tau [33]–[36], MAP2c [37] and MAP1B [38]. This ability of MAPs to link the two cytoskeletons likely contributes to specific morphogenetic events that require actin/microtubule coupling, such as neurite turning, branching and retraction.

In addition to their cytoskeletal affinities, MAP6 proteins have been reported to associate with the Golgi through palmitoylation in heterologous cells [5]. Here, we demonstrated that MAP6d1 and MAP6-N are palmitoylated in vivo in neurons. We examined palmitoylation events using a battery of MAP6d1 mutants, and we found that three cysteine residues (Cys 5, 10 and 11) located in the N-terminus of the protein can be palmitoylated. In heterologous cells, palmitoylated MAP6d1 mainly localizes to the Golgi and can also be found at the plasma membrane. MAP6d1 was not detected at the endoplasmic reticulum. Such selective subcellular localization of palmitoylated MAP6d1 likely relies on distinct palmitoyl transferases (DHHC proteins) known to be located and active in several subcellular compartments, including the endoplasmic reticulum, the Golgi, the plasma membrane [17] and some mobile dendritic vesicles in neurons [39]. Indeed, we showed that MAP6d1 proteins are substrates for DHHC-2, 3, 7 and 15, which are mainly associated with the Golgi, and DHCC-20 and 21, which are present at the plasma membrane. Palmitoylation at the plasma membrane is particularly relevant in neurons, where it is believed to regulate many synaptic proteins, including glutamate receptors and synaptic vesicle proteins [30]. In this respect, it is interesting to note that DHHCs that act on MAP6d1 palmitoylate several other synaptic proteins, including PSD95 and SNAP25 [29]. Several pieces of data suggest that MAP6 proteins localize to both pre- and post-synaptic compartments. MAP6s have been identified in the proteomes of post-synaptic densities (PSDs) [40]–[43] and synaptic vesicles [40], [44]. Phosphorylated forms of MAP6 proteins partially co-localize with synaptic markers, such as Homer or synapsin, in cultured neurons [15]. Direct interaction between MAP6 and SNAP25 proteins has been described [45]. We propose that the reversible palmitoylation of synaptic MAP6 proteins, in addition to other synaptic proteins, may play a role in regulating synaptic transmission and plasticity.

The microtubule-related proteins of the stathmin family (SCG10, SCLIP and RB3) are crucially involved in neuronal differentiation and maturation. They localize to the Golgi complex via palmitoylation as well as to vesicles along axons and dendrites [46]. Interestingly, stathmin and MAP6 proteins are both substrates for the DHHC-2, 3, 7 and 15 enzymes. These data suggest that stathmin and MAP6s form macromolecular complexes on vesicles along developing dendrites. These complexes are crucial for neuronal differentiation [46]. Subcellular localization of stathmins and MAP6 proteins on the Golgi (or vesicles) may be crucial to the promotion of some of their microtubular functions, such as local control of microtubule dynamics.

MAP6d1 localization at the mitochondria was observed, consistent with published mitochondrial proteomes that include MAP6 proteins [47], [48]. Interestingly, the N-terminal domains of stathmin proteins are required for their targeting to mitochondria, independently of palmitoylation, as shown for MAP6d1 [49]. At physiological levels, MAP6 proteins may be involved in glutamine metabolism, as MAP6 deletion affects glutamine levels in the brain [50] and as MAP6 interacted with Bmcc1s [51], which regulate the kidney phosphate-activated glutaminase, a key mitochondrial enzyme responsible for the conversion of glutamine to glutamate in neurons [52]. Other MAPs have been shown to directly interact with mitochondrial proteins, including LC3/MAP1b, which binds to cardiolipin [53], or Tau, which interacts with VDAC, the mitochondrial fission protein Drp1 [54], [55] and the adenine nucleotide translocator 1 (ANT-1)[56]. Abnormal Tau interactions with mitochondrial proteins have been reported to be causal in mitochondrial dysfunction in Alzheimer's diseases [54]–[57], and abnormal LC3/MAP1b interaction with mitochondria resulted in mitophagy in Parkinson disease models [53]. Future studies should identify specific mitochondrial functions of MAP6s in neurons and possible mitochondrial deficiencies in MAP6-null cellular models.

Our results also demonstrate that MAP6 proteins multimerize in a microtubule-binding module Mn3 dependent manner. This finding questions about cellular mechanisms responsible for regulating shifts of MAP6 from microtubules to multimers. Because MAP6 proteins contain three Mn domains, we speculate that MAP6 binds to microtubules via its Mn1 and Mn2 domains and multimerizes through its Mn3 domains, thereby contributing to microtubule bundling. Indeed, multimerization has been reported for other MAPs, such as Tau. Whether this multimerization is dependent on microtubule interaction is still controversial [58], [59]. Additional studies are needed to clarify the physiological consequences of MAP multimerization in the context of neuronal development and function.

**Figure 10:**
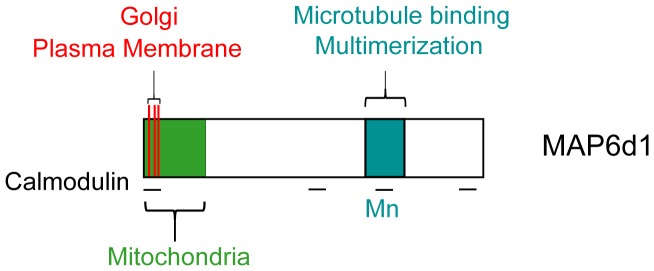
Schematic representation of MAP6d1 protein. MAP6d1 domains involved in association with the Golgi, plasma membrane, mitochondria and calmodulin are shown. Also shown is the Mn3 domain, which is involved in microtubule binding and multimerization.

The intrinsic biochemical properties of MAP6, summarized in Fig. 10, raise questions concerning how and when MAP6 proteins shuttle between microtubules, actin, membranous compartments and multimers in neurons. Can they bind microtubules and actin at the same time? Can MAP6 proteins at the Golgi or plasma membrane promote microtubule nucleation? Can MAP6 multimers promote bundling or nucleation of microtubules? In-depth structural and biochemical studies should provide answers to these questions and hint at the role of MAPs in neuronal development and plasticity.

## Supporting Information

S1 File (PDF)
