# Supplementary material for: Non-Microtubular Localizations of Microtubule-Associated Protein 6 (MAP6)
Source: PLoS One. 2014 Dec 19;9(12):e114905. doi: 10.1371/journal.pone.0114905 (PMC4272302; doi:10.1371/journal.pone.0114905)
Supplement: S1 File — (PDF) [file pone.0114905.s001.pdf]

Table 1: Subcellular localization of MAP6d1 and MAP6d1 mutants (raw data)

Constructs	# cells with microtubule localization	# cells with Golgi localization	# cells with plasma membrane localization	# cells with mitochondria localization	# cells with other localization	# cells total
MAP6d1	6	188	12	135	10	221
MAP6d1-GGG	38	0	0	171	78	287
MAP6d1-Δ2-34	37	0	0	0	163	200
